# Prevalence and Determinants of Overweight and Obesity Among Adolescent School Children of South Karnataka, India

**DOI:** 10.4103/0970-0218.62587

**Published:** 2010-01

**Authors:** M Shashidhar Kotian, Ganesh Kumar S, Suphala S Kotian

**Affiliations:** Department of Community Medicine, Kasturba Medical College, Mangalore, India; 1 Department of Preventive and Social Medicine, JIPMER, Puducherry, India

## Introduction

At present the potential public health issue that is emerging is the increasing incidence of childhood obesity in developing countries, and the resulting socioeconomic and public health burden that will be faced by these countries in the near future. The prevalence is higher in the urban than in the rural areas. Many studies have shown that the prevalence of overweight among adolescents varies between 10 and 30%.(1–11) Prevalence varies within the country because of differences in the lifestyle, mainly in the dietary patterns, and physical activity. In addition to this urbanization and industrialization are the main culprits for the increase in the prevalence of childhood obesity. No published literature can be found in this part of the country to assess the prevalence and determinants of obesity among adolescents. Studies of such a nature will be useful tools in planning and developing appropriate intervention methods. In this context, the present study has been conducted to estimate the prevalence and determinants of overweight and obesity among school children aged between 12 and 15 years, in a city in South Karnataka.

## Materials and Methods

This was a school-based, cross-sectional study carried out over a period of four months, from January to April 2007. The sample size was estimated for infinite population by using the formula 4pq/d^2^ where prevalence was taken as 10%.(1) The required precision of the estimate (d) was set at 20%. Using the above-mentioned formula, the sample size was estimated to be 900. After adding the non-response error of 10%, an additional 100 subjects were included. Thus, 1000 subjects were selected for this study. We adopted a multistage stratified random sampling procedure. For the selection of schools, a list of all schools was obtained from the school authorities of the district education office. First, six schools were selected by a simple random technique. Probability, proportional to the size sampling technique was used to select the sample from each school.

The subjects were adolescents, 12 to 15 years of age, in the city of Mangalore, Karnataka, a Southern State of India. After reaching the concerned school, the classes were selected randomly from each grade. Students were selected from each class by the simple random technique, using the students' register, till the desired sample from each class was met. It was assumed that from each institution, at least 50 subjects would be recruited from each class. If a designated student could not be contacted or was not cooperative during the three separate visits, the subject was considered as a non-respondent. Trained investigators weighed all of the adolescents without shoes and heavy clothing, using an electronic weighing scale with an error of ±100 g. The weighing scale was regularly checked with known standard weights. A portable anthropometric rod was used for measuring the height, with an error to the nearest of 0.1 cm, using standard procedures.(12) The International Obesity Task Force references were used to define overweight and obesity in this study.(13) Information was collected on physical activity, which included the mode of transport used to go to school and physical activities such as participation in sports and games, aerobic physical exercises, and frequency and duration of participation in household activities. Time spent in watching television and playing computer and video games were also noted. Diet preferences for chocolate, biscuits, chips, and colas were taken into consideration. Adolescents were categorized into two groups namely overweight (≥ wighty-fifth percentile) and obese (≥ ninety-fifth percentile). The socioeconomic status was assessed based on the Kuppuswamy classification. Univariate and Multiple Logistic Regression analyses were carried out. For all statistical tests, *P* < 0.05 was taken as the significant level.

## Results

A total of 900 adolescents in the age group of 12 to 15 years were analyzed. Out of these 461 (51.2%) subjects were males. The mean BMI of the sample was 17.3 kg/m^2^.

The overall prevalence of overweight among adolescents was 9.9% and obesity was 4.8% [Table 1]. The prevalence of overweight was 9.3% among boys and 10.5% among girls; 5.2 and 4.3% were obese, respectively. However, according to the Body Mass Index cut off values, 23.9% (215) were underweight (< 18.5), 60.6% (546) were normal (18.5 – 24.9), 11.4% (103) were overweight (25 – 29.9), and 4% (36) were obese (30 and above). A multivariate logistic regression analysis revealed that the risk of overweight was two times higher among the adolescents of high SES, 21 times higher among those participating < two hour/week in any type of physical activity, 7.3 times higher among those who reported watching television and playing games on the computer for ≥ four hours/day, and 5.6 times higher among those who ate chocolates daily in addition to a normal diet [Table 2].

**Table 1:** Prevalence of overweight and obesity according to its determinants N = 900

Determinants	Number of subjects examined	Number of overweight subjects (Prevalence in %)	Number of obese subjects (Prevalence in %)	χ^2^, *P* value
Sex				
Boys	461	43 (9.3)	24 (5.2)	0.67, 0.72
Girls	439	46 (10.5)	19 (4.3)	
Age				
12	177	17 (9.6)	8 (4.5)	0.37, 0.999
13	255	26 (10.2)	12 (4.7)	
14	242	25 (10.3)	11 (4.5)	
15	226	21 (9.3)	12 (5.3)	
Socioeconomic status				
Grade 1	68	11 (16.2)	9 (13.2)	21.714, 0.001*
Grade 2	170	18 (10.6)	12 (7.1)	
Grade 3	510	44 (8.6)	19 (3.7)	
Grade 4	152	16 (10.5)	3 (2.0)	
Exercise duration(in hrs)				
<1	627	69 (11)	33 (5.3)	3.32, 0.51
1-2	213	19 (8.9)	10 (4.7)	
>2	60	1 (1.7)	0 (0)	
TV, Computer watching in hrs				
<2	436	23 (5.3)	4 (0.9)	77.33, 0.001*
2-4	331	40 (12.1)	19 (5.7)	
>4	133	26 (19.5)	20 (15)	
Diet preferences				
Normal	710	52 (7.3)	14 (2.0)	98.35, 0.001*
+ chocolate	108	19 (17.6)	13 (12.0)	
+ chocolate + biscuits	18	4 (22.2)	3 (16.7) +	
+ chocolates + chips	41	9 (22.0)	8 (19.5)	
+cola	22	5 (22.7)	5 (22.7)	

* *P* value less than 0.05 is considered as significant.

**Table 2:** Correlates of overweight and obesity; Multiple logistic regression analysis

Variables	Adjusted odds ratio	95% CI	*P* value
Socioeconomic status			
Grade 4	-	-	-
Grade 1	2.09	1.1–4.0	0.02*
Grade 2	1.64	0.8–3.3	0.17
Grade 3	0.99	0.42–2.32	0.98
TV, Computer watching in hrs			
<2	-	-	-
2-4	2.48	1.3–4.6	0.004*
>4	7.3	3.6–14.66	0.001*
Diet preferences			
Normal	-	-	-
+ chocolate	5.58	2.1–14	0.001*
+ chocolate + biscuits	1.3	0.5–3.8	0.6
+ chocolates + chips	1.02	0.24–4.27	0.98
+ cola	0.96	0.3–3.0	0.95
Physical activity duration in hrs			
>2	-	-	-
1-2	1.56	0.94–2.6	0.09
<1	21.09	2.77–166.8	0.003*

* *P* value less than 0.05 is considered as significant

## Discussion

The overall prevalence of overweight adolescents among the urban group was found to be 9.9%, which was consistent with a recent study.(2) However, the National Nutrition Monitoring Bureau surveys in 2002, in rural areas, reported the prevalence of as little as 0.6%.(3) A similar study done in Hyderabad showed that the prevalence of overweight was 7.2% among the 12 to 17 year age group.(4) Although, some other studies done in India showed a higher prevalence of overweight and obesity.(5–8) A study in Delhi on affluent school children showed the prevalence of obesity to be 7.4%.(9) Another study among affluent girls in Delhi reported the prevalence of obesity and overweight to be 5.3 and 15.2%, respectively.(10) Similar studies had been conducted to assess the prevalence of overweight and obesity in India and the results are comparable to our study, with respect to the prevalence of obesity.(11,14) A study done in USA during 2001–2002 showed the prevalence of overweight and obesity as 31.5 and 16.5%, respectively, for the 6 to 19 year age group.(15) The widely differing prevalence of overweight and obesity was due to the definitions used, age group and sex taken for the study, uniformity of selection of the sample, area selected, and the methodology used for the survey.

Overweight and obesity were marginally higher in the pubertal age groups of 13 to 15 years, perhaps because of increased adipose tissue and overall body weight in children during puberty. One of the major reasons for childhood obesity was watching television or using computers as shown by another study.(4,16) A clear socioeconomic gradient in the prevalence of overweight and obesity was observed in this study, which was consistent with other studies.(4,5,17) The results revealed that regular physical activity was an important factor in reducing the prevalence of overweight and obesity, which was consistent with other studies.(4,5)

We could not interview 100 non-respondents because of their non-availability during our field visits. As the proportion of non-respondents was within the limits, we expected only a minimal effect on our prevalence estimate. A normal diet survey was not performed because of feasibility constraints. It was concluded that the overall prevalence of overweight was 9.3% among boys and 10.5% among girls; 5.2 and 4.3% were obese, respectively. The prevalence of overweight was higher among the adolescents of the high socioeconomic status group, who had physical activity of < one hour/day, watched television ≥ 4 hours/day, and ate chocolates daily. Action needs to be taken to curb the problem of obesity among adolescents. Public health interventions at the individual and policy-making levels need to be instigated at the earliest, to tackle this problem in the country.

## References

[1] Subramanya V, JayaShree R, Rafi M (2003). Prevalence of overweight and obesity in affluent adolescent girls in Chennai in 1981 and 1998. Indian Pediatr.

[2] Aggarwal T, Bhatia RC, Singh D, Sobti PC (2008). Prevalence of obesity and overweight in affluent adolescents from Ludhiana, Punjab. Indian Pediatr.

[3] National Nutrition Monitoring Bureau (2002). Diet and nutritional status of rural population national institute of nutrition.

[4] Laxmaiah A, Nagalla B, Vijayaraghavan K, Nair M (2007). Factors affecting prevalence of overweight among 12 to 17 year-old urban adolescents in Hyderabad, India. Obesity (Silver Spring).

[5] Ramachandran A, Snehalatha C, Vinitha R, Thayyil M, Kumar CK, Sheeba L (2002). Prevalence of overweight in urban Indian adolescent school children. Diabetes Res Clin Pract.

[6] Chatterjee P (2002). India sees parallel rise in malnutrition and obesity. Lancet.

[7] Kaur S, Kapil U, Singh P (2005). Pattern of chronic diseases amongst adolescent obese children in developing countries. Curr Sci.

[8] Khadilkar VV, Khadilkar AV (2004). Prevalence of obesity in affluent school boys in Pune. Indian Pediatr.

[9] Kapil U, Singh P, Pathak P, Dwivedi SN, Bhasin S (2002). Prevalence of obesity amongst affluent adolescent school children in Delhi. Indian Pediatr.

[10] Mehta M, Bhasin SK, Agrawal K, Dwivedi S (2007). Obesity amongst affluent adolescent girls. Indian J Pediatr.

[11] Kaneria Y, Singh P, Sharma DC (2006). Prevalence of overweight and obesity in relation to socio economic conditions in two different groups of school age children of Udaipur city (Rajastan). J Indian Assoc Community Med.

[12] Jelliffee DB (1988). Assessment of nutritional status of the community.

[13] Cole TJ, Bellizzi MC, Flegal KM, Dietz WH (2000). Establishing a standard definition for child overweight and obesity: International survey. BMJ.

[14] Sidhu S, Marwah G, Prabhjot (2005). Prevalence of overweight and obesity among the affluent school children of Amritsar, Punjab. Coll Antropol.

[15] Hedley AA, Ogden CL, Johnson CL, Carroll MD, Curtin LR, Flegal KM (2004). Prevalence of overweight and obesity among US children, adolescents and adults. 1999-2002. JAMA.

[16] Eisenmann JC, Bartee RT, Wang MQ (1999). (2002) Physical activity, TV viewing, and weight in US youth: Youth risk behaviour survey. Obestet Res.

[17] Kasmini K (1997). Prevalence of overweight and obesity among school children aged between 7–16 years amongst the 3 major ethnic groups in Kuala Lampur, Malaysia. Asia Pac J Clin Nutr.

